# Response to the letter to the editor: Lansoprazole interferes with fungal respiration and acts synergistically with amphotericin B against multidrug-resistant Candida auris*

**DOI:** 10.1080/22221751.2024.2396869

**Published:** 2024-08-28

**Authors:** Ehab A. Salama, Yehia Elgammal, Aruna Wijeratne, Nadia A. Lanman, Sagar M. Utturkar, Atena Farhangian, Jianing Li, Brigitte Meunier, Tony R. Hazbun, Mohamed N. Seleem

**Affiliations:** a Department of Biomedical Sciences and Pathobiology, Virginia-Maryland College of Veterinary Medicine, Virginia Polytechnic Institute and State University, Blacksburg, VA, USA; b Center for One Health Research, Virginia Polytechnic Institute and State University, Blacksburg, VA, USA; c Department of Biomedical Sciences and Pathobiology, Virginia-Maryland College of Veterinary Medicine, Virginia Polytechnic Institute and State University, Blacksburg, VA, USA; d Center for One Health Research, Virginia Polytechnic Institute and State University, Blacksburg, VA, USA; e Department of Biochemistry and Molecular Biology, Indiana University School of Medicine, Indianapolis, IN, USA; f Purdue Institute for Cancer Research, Purdue University, West Lafayette, IN, USA; g Department of Comparative Pathobiology, Purdue University, West Lafayette, IN, USA; h Purdue Institute for Cancer Research, Purdue University, West Lafayette, IN, USA; i Department of Medicinal Chemistry and Molecular Pharmacology, Purdue University, West Lafayette, IN, USA; j Department of Medicinal Chemistry and Molecular Pharmacology, Purdue University, West Lafayette, IN, USA; k Université Paris-Saclay, CEA, CNRS, Institute for Integrative Biology of the Cell (I2BC), Gif-sur-Yvette, France; l Purdue Institute for Cancer Research, Purdue University, West Lafayette, IN, USA; m Department of Medicinal Chemistry and Molecular Pharmacology, Purdue University, West Lafayette, IN, USA; n Department of Biomedical Sciences and Pathobiology, Virginia-Maryland College of Veterinary Medicine, Virginia Polytechnic Institute and State University, Blacksburg, VA, USA; o Center for One Health Research, Virginia Polytechnic Institute and State University, Blacksburg, VA, USA

## Reply

We thank Zhu et al. [1] for showing interest in our recent work (Lansoprazole interferes with fungal respiration and acts synergistically with amphotericin B against multidrug-resistant *Candida auris*) [2]. We have carefully reviewed their comments/suggestions. However, there may have been a misunderstanding of our study’s objective. We understand that our study may have raised some questions, and we appreciate the opportunity to clarify our objectives and findings. Our study highlighted the synergistic interaction between amphotericin B and lansoprazole against the highly resistant fungus, *Candida auris*. The work was supported by mechanistic studies and validated using an animal model.

We emphasize that the concerns raised by Zhu et al. [1] were explicitly mentioned in our limitations section. Furthermore, we clarify that our study is not a clinical trial and only focused on specific aspects of combination therapy without addressing long-term clinical implications in animals and humans.

Although we outlined and discussed the important limitations, we had limited space to fully expand every aspect of these limitations. We had hoped to see constructive criticism that would provide more in-depth insights, enrich the outcomes of our study, and offer readers an additional scientific perspective.

In our study, we have clearly discussed and addressed the following limitations:

1. We acknowledged that the elevated dose of lansoprazole used in this study is a limitation. However, this provided proof of concept for the clinical potential of combination therapy and emphasized the role of cytochrome bc1 as a potential antifungal target. Moreover, a similar dose of lansoprazole sulfide was successfully used in mice for treating a tuberculosis infection [3].
2. The need for comprehensive toxicological study was clearly mentioned in the limitation. Nevertheless, we supported our data with an efficacy animal study that showed no signs of toxicity during the treatment period.
3. The instability of lansoprazole and the identification of the specific metabolite(s) that could be the key reason for the biological activity of lansoprazole were also clearly stated.

These were the main criticisms outlined in the letter and are merely reiterations of what we have clearly stated in the manuscript, albeit in a limited manner due to space limitations.

Other points we want to clarify:

1. The letter’s authors suggested a detailed treatment plan, assessment of the long-term antifungal efficacy of the combination therapy, and the pharmacokinetic/metabolism of lansoprazole in humans. These aspects are entirely outside the scope of our study, which is not intended as a clinical trial.
2. We agree that investigating and ruling out other published potential mechanisms contributing to the efficacy of lansoprazole would be beneficial. However, the reference provided by Zhu et al. [1] does not include any mechanistic study and only pointed out some potential mechanisms in the discussion section [4], leaving us without sufficient information to pursue a mechanistic study of lansoprazole as recommended by the authors.
3. The letter referenced other works that do not appear to be directly relevant. For instance, it cited a paper on the long-term durability of the treatment [5], which addresses antitumour activity and is not applicable to our research.

Despite its limitations, our study opens a new avenue for identifying scaffolds, adding more chemical diversity to the almost dry antifungal pipeline. We still believe that our study provides robust data on the synergy between amphotericin B and lansoprazole. We thank everyone who shows interest in our research and hope that these discussions benefit readers and contribute to the advancement of science.
